# The Prevalence of Problem Gambling and Gambling Disorder Among Homeless People: A Systematic Review And Meta-Analysis

**DOI:** 10.1007/s10899-022-10140-8

**Published:** 2022-07-19

**Authors:** Karl Deutscher, Stefan Gutwinski, Felix Bermpohl, Henrietta Bowden-Jones, Seena Fazel, Stefanie Schreiter

**Affiliations:** 1 Department of Psychiatry and Neurosciences, Charité – Universitätsmedizin Berlin, Corporate Member of Freie Universität Berlin and Humboldt-Universität zu Berlin, and Berlin Institute of Health, 10117 Berlin, Germany; 2 National Problem Gambling Clinic, London, SW5 9BH UK; 3 Department of Psychiatry, University of Cambridge, Cambridge, CB2 8AH UK; 4 Faculty of Brain Sciences, University College London, London, WC1E 6BT UK; 5 Department of Psychiatry, University of Oxford, Oxford, OX3 7JX UK; 6 Oxford Health NHS Foundation Trust, Oxford, UK

**Keywords:** Homelessness, Problem gambling, Gambling disorder, Prevalence, Meta-analysis, Systematic review

## Abstract

Gambling problems are often associated with homelessness and linked to elevated psychiatric morbidity and homelessness chronicity. We performed a systematic review and meta-analysis on prevalence rates of problem gambling (PG) and gambling disorder (GD) in homeless people. Following PRISMA guidelines, we searched databases Medline, Embase and PsycINFO from inception of databases to 4th may 2021. We included studies reporting prevalence estimates on clinical gambling problems in representative samples of homeless people based on standardized diagnostics. Risk of bias was assessed. A random effects meta-analysis was performed, and subgroup analyses based on methodological characteristics of primary studies were conducted. We identified eight studies from five countries, reporting information on 1938 participants. Prevalence rates of clinically significant PG and GD ranged from 11.3 to 31.3%. There was evidence for substantial heterogeneity with I^2^ = 86% (95% CI 63–97%). A subgroup of four low risk of bias studies displayed a significantly lower results ranging from 11.3 to 23.6%. Additionally, high rates of subclinical problem gambling were reported (11.6–56.4%). At least one in ten homeless persons experiences clinically significant PG or GD. Social support and health care services for the homeless should address this problem by implementing models for early detection and treatment.

**Supplementary Information:**

The online version contains supplementary material available at 10.1007/s10899-022-10140-8.

## Introduction

Homelessness is widely recognized as a severe social and public health issue on a global scale (E/CN.^5^/, 2020/3, 2020). Approximately 550,000 individuals are currently affected by homelessness in the US (Henry et al., 2018), 700,000 in the EU and UK (Serme-Morin et al., 2020). Homeless populations are burdened with disproportionately high prevalence rates of mental disorders (Gutwinski et al., 2021). Substance use disorders, in particular, are common, with around a third with alcohol use disorder and a quarter with drug use disorder (Gutwinski et al., 2021). Substance use is considered a major risk factor for the onset and chronicity of homelessness (Shelton et al., 2009; North et al., 1998; Calvo et al., 2020), and has been identified as one of the most important contributors to the significantly increased mortality in homeless people (Nielsen et al., 2011; Beijer et al., 2011).

In recent years, gambling disorder has been increasingly recognized as an addictive disorder similar to substance-based addictions due to its similar personality-related, neurobiological and clinical features, resulting in DSM-5 and ICD-11 reclassifying it in the same category as substance-related disorders (Kim & Hodgins, 2019). These similarities, in conjunction with the major impact of substance use disorders on people experiencing homelessness, suggest that rates of gambling problems might also be increased within this population.

A pattern of gambling behaviour marked by high levels of persistence or recurrence and consequential distress and functional impairment can constitute a pathology. The latest iterations of both DSM and ICD refer to clinically relevant gambling problems as “gambling disorder” (GD) (Diagnostic & Statistical, 2013; International, 2019). The broader term “problem gambling” (PG) is often used to additionally include subclinical levels of problematic gambling (Weinstock et al., 2017).

Like homelessness, gambling problems have potentially extensive negative effects. People experiencing PG/GD report significantly decreased quality of life in comparison to people who are not affected (Scherrer et al., 2005), often mediated by the frequently occurring financial decline (Grant et al., 2010). PG/GD is associated with high rates of psychiatric comorbidity (Lorains et al., 2011). A recent nationwide register study from Sweden determined the rate of psychiatric comorbidities of patients treated at GD with 73%, with anxiety disorders, affective disorders and substance use disorders as the most common diagnoses (Håkansson et al., 2018). The overall mortality ratio and specifically suicide mortality were shown to be considerably elevated (Karlsson & Håkansson, 2018). Consequently, people experiencing homelessness and gambling problems at the same time might face particularly increased health risks. In addition, financial difficulties which frequently result from gambling problems might elevate the risk of homelessness chronicity (Kostiainen, 2015).

While prevalence estimates on gambling problems in general populations across the world are marked by substantial heterogeneity, partially due to large differences in methodology and definitions between surveys (Calado & Griffiths, 2016), there is some consensus that marginalized populations are particularly affected: Increased prevalence rates are found in ethnic minorities, inhabitants of socioeconomically disadvantaged neighbourhoods and people experiencing homelessness (Hahmann et al., 2020). Furthermore, large population-based surveys demonstrated a significant association between PG/GD and homelessness (Edens & Rosenheck, 2012; Moghaddam et al., 2015).

Precise estimates on the prevalence of PG/GD among the homeless are important to inform service development and evidence-based policy. Detecting and addressing PG/GD might be a key factor to achieve more positive outcomes in many cases of practical service work with people affected by homelessness that has often been overlooked. Several publications have narratively reviewed literature on the prevalence of PG/GD in people experiencing homelessness (Hahmann et al., 2020; Sharman, 2019; Stephanie et al., 2018), but there are no systematic reviews to our knowledge.

### Aims of the Study

The objective of this article is to systematically review the prevalence of PG and GD in homeless populations. We aim to compile a complete overview on the scientific evidence, to provide quantitative synthesis via meta-analytical models and to investigate potential sources of heterogeneity through subgroup analyses.

## Methods

The protocol for this review was registered at PROSPERO (registration ID CRD42021233670). The authors followed the PRISMA statement (Preferred Reporting Items for Systematic Reviews and Meta-Analyses (Moher et al., 2010), see Online Resource 1).

### Eligibility Criteria

We sought to identify primary studies that could provide prevalence estimates of PG/GD in homeless samples in online scientific data bases. Studies had to meet the following inclusion criteria to be included in the review:

A) A prevalence estimate (12-months prevalence or lifetime prevalence) of PG/GD was determined.
B) A separate sample of exclusively and reliably homeless individuals was included.
C) Participants were individually examined for PG/GD using a standardized diagnostic instrument.

Studies which sampled specific subpopulations not representative for the homeless population as a whole (i.e., exclusively homeless persons with mental disorders, selected age bands etc.) were to be excluded.

### Systematic Search

In order to identify eligible records, Medline via PubMed, Embase via OvidSP and PsycInfo via EBSCOhost were searched by specifically formulated entries containing key words associated with homelessness and gambling (see Online Resource 2). Additionally, we screened the reference lists of included and other major publications for relevant studies. No restrictions on publication language were applied. Records published between the inception of data bases and 4th of May 2021 were included. Search results were independently scanned for eligible articles by two researchers. Differences in screening results were resolved in discussion.

### Data Extraction

Data from included studies for study location, years of study conduct, assessment used in diagnosing PG/GD, recruitment strategy, sampling method, information regarding psychiatric morbidity, mean age, gender distribution, sample size and number of detected cases of PG/GD was extracted. In cases of missing information, authors of primary studies were contacted to provide additional data.

Special attention was paid to diagnostic instruments used to assess PG/GD. A full version of each inventory was acquired by web search to examine their methodological characteristics.

Included studies were evaluated regarding risk of bias by a standardized assessment tool (Hoy et al., 2012). Each item was individually evaluated. For the summary item, we rated studies as low risk of bias when eight or more items out of 10 items indicated “low risk”, any others as moderate risk of bias. Both data extraction and quality evaluation were carried out by two researchers independently from one another, discussing diverging results afterwards.

### Quantitative Analysis

Prevalence estimates corresponding to clinically relevant PG/GD were entered into a meta-analytical model. All statistical analyses were carried out in R, version 4.0.4 (Bates et al., 2021), using the package ‘metafor’, version 2.4-0 (Viechtbauer, 2010). A Freeman Turkey double arcsine transformation was applied to the prevalence estimates (Freeman & Turkey, 1950), so variance instability could be avoided (Barendregt et al., 2013). We calculated random effects models, estimating the variance by the Paule-Mandel method (Paule & Mandel, 1934). A 95% Wald-type confidence interval (CI) was computed around the random effects weighted mean, as well as a 95% prediction interval (PI), the latter by a method which accounts for the model variance to be an estimated value ((Higgins et al., 2009), expression 12). A Q-test for heterogeneity was conducted and the I^2^ statistic was computed (Ioannidis et al., 2007).

For a secondary analysis, we constructed a three-level meta-analytic model for the same data, using the ‘metafor::rma.mv’ function. The underlying assumption was that the 12-months prevalence rates and lifetime prevalence rates included in the analysis might constitute slightly different effect sizes, introducing a dependency (study estimates being “nested” within the prevalence types) which might lead to an underestimation of the model heterogeneity. A three-level model has an additional layer integrated into its structure to account for clustered data like this (Cheung, 2014). The fit of this secondary model was compared to the primary one with the ‘metafor::anova’ function by the Akaike criterion corrected for small samples (AIC_C_).

To examine the impact of methodological characteristics, we conducted subgroup analyses, grouping studies by prevalence type (lifetime vs. past-year prevalence), PG/GD criteria (DSM-based vs. not DSM-based), overall risk of bias (low risk of bias vs. moderate risk of bias), sample mean age (> 45 years vs. < 45 years) and proportion of female participants (> 20% vs. < 20%). Random effects weighted means and 95% CIs were calculated for each group separately and the between-groups heterogeneity was assessed by a Q-test.

## Results

### Study Selection

**Fig. 1:**
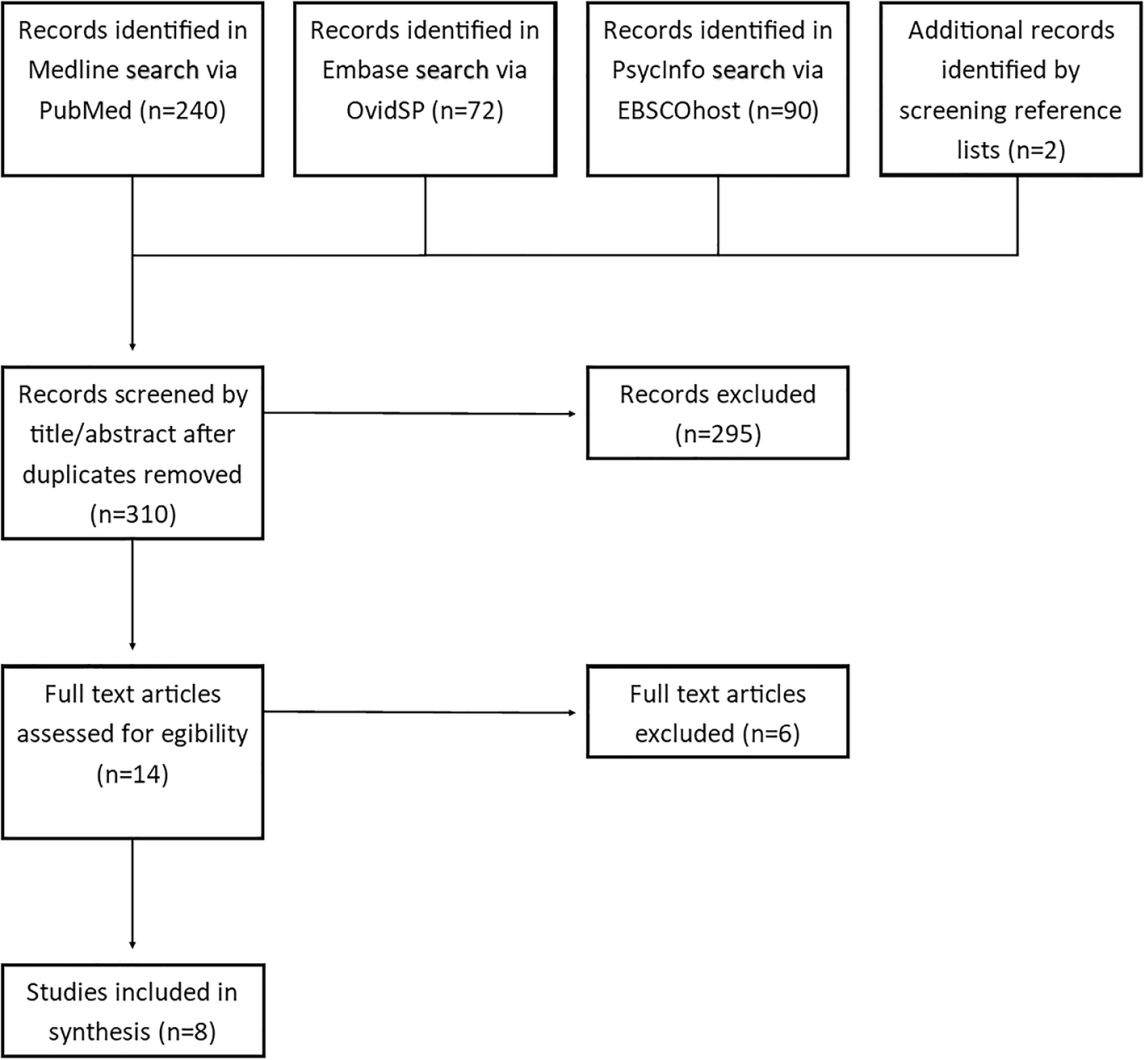
PRISMA Flow Chart

The database search entries returned 310 distinct records after duplicates were removed (see Fig. 1). Eight publications were found to be eligible (Gattis & Cunningham-Williams, 2011; Matheson et al., 2014, 2021; Nower et al., 2015; Pluck et al., 2015; Sharman et al., 2015, 2016; Wieczorek et al., 2019) (for information on articles rejected in full-text screening see Online Resource 3). They were published between 2011 and 2021 and conducted in five different countries: Japan (Pluck et al., 2015), Poland (Wieczorek et al., 2019) and two each in Canada (Matheson et al., 2014, 2021), the US (Gattis & Cunningham-Williams, 2011; Nower et al., 2015) and the UK (Sharman et al., 2015, 2016).

### Study Characteristics

Data on a total of 1938 homeless individuals was included by these surveys. For 1527 (77.0%) participants, information on gender was provided, identifying 1179 (77.2%) as male and 348 (22.8%) as female (Gattis & Cunningham-Williams, 2011; Matheson et al., 2021; Nower et al., 2015; Pluck et al., 2015; Sharman et al., 2015, 2016; Wieczorek et al., 2019). A mean age of 46 years (Nower et al., 2015; Pluck et al., 2015; Sharman et al., 2015, 2016; Wieczorek et al., 2019) was reported in 1213 (61.1%) participants. See Table 1 for additional study characteristics. In quality assessment, four studies were rated as low risk of bias and four as moderate risk of bias (see Online Resource 4).

**Table 1:** Study Characteristics

Study	Location	Period of data collection	Sampling	Participation Rate	Sample size	Mean age	Proportion of female participants
Gattis, 2011^a^	St. Louis, MO, USA	2003–2004	Community advertising and telephone screening	n.r	48	n.r	33%
Matheson, 2014	Toronto, Canada	2013	Time/location sampling at different services provided by a community organization	n.r	264	47	n.r
Matheson, 2021	Hamilton and Toronto, Canada	2019	Time/location sampling at shelters and drop-in programs provided by multiple organizations	42%	162	n.r	100%
Nower, 2015	St. Louis, MO, USA	n.r	Random or representative sampling from shelters and street locations	92%	275	41	27%
Pluck, 2015	Tokyo, Japan	n.r	Clients of a charity organization for the homeless	94%	16	52	0%
Sharman et al., 2015	London, UK	2012	Sampling from shelters, hostels and day centres across one city borough	–^b^	456	42	7%
Sharman, 2016	London, UK	2014	Sampling from shelters, hostels and day centres across one city borough	–^b^	72	41	13%
Wieczorek et al., 2019	Warsaw, Poland	2015–2016	Rehabilitation shelters and night shelters of the city	–^b^	690	50	10%

^a^Sample characteristics described exclusively for the “unstable housing” sub-sample

^b^The sampling process of this study did not allow for a response rate to be determined

*M.O*: Missouri, *n.r:* not reported

Five studies utilized instruments for the diagnosis of PG/GD according to definitions by different versions of DSM criteria which refer to it as “pathological gambling” (Gattis & Cunningham-Williams, 2011; Matheson et al., 2014, 2021; Nower et al., 2015; Pluck et al., 2015). The Computerized Gambling Assessment Module (C-GAM) (Cunningham-Williams et al., 2003), the NORC Diagnostic Screen for Disorders (NODS) (Hodgins, 2004) and an Assessment of Gambling Problems as proposed by Ricketts & Bliss (Ricketts & Bliss, 2003) are based on DSM-IV criteria for “pathological gambling”, the South Oaks Gambling Screen (SOGS) on DSM-III and DSM-III-R criteria (Lesieur & Blume, 1987). Another three studies (Sharman et al., 2015, 2016; Wieczorek et al., 2019) assessed gambling behaviour by the Problem Gambling Severity Index (PGSI) which does not relate to any fixed set of diagnostic criteria directly since it was conceived primarily to serve as a continuous scale for problem gambling severity (Ferris & Wynne, 2001).

Prevalence rates at lifetime were reported by four studies (Gattis & Cunningham-Williams, 2011; Matheson et al., 2014, 2021; Nower et al., 2015), while another four provided 12-month prevalence rates (Pluck et al., 2015 Sep; Sharman et al., 2015; Sharman et al., 2016 Jun; Wieczorek et al., 2019).

### Prevalence of Problem Gambling/Gambling Disorder

Estimates of PG/GD prevalence ranged between 11.3 and 31.3% (Gattis & Cunningham-Williams, 2011; Matheson et al., 2014; Nower et al., 2015; Pluck et al., 2015; Sharman et al., 2015, 2016; Wieczorek et al., 2019). Additionally, six studies provided rates of subthreshold PG, indicating that additionally between 11.6 and 56.4% of participants displayed different degrees of subclinical at-risk gambling behaviour (Gattis & Cunningham-Williams, 2011; Matheson et al., 2014, 2021; Nower et al., 2015; Sharman et al., 2015, 2016) (see Table 2).

**Table 2:** Assessments and Results

Study	Instrument	Underlying criteria	Interpretive categories	Rate	Prevalence Type
Gattis, 2011	C-GAM	DSM-IV pathological gambling	1–4/10 criteria = Subthreshold gambling	56.4%	Lifetime prevalence
			** ≥ 5/10 criteria = pathological gambling disorder**	**27.1%**	
Matheson, 2014	NODS	DSM-IV pathological gambling	1–2/10 score = at-risk gambling (mild subclinical risk)	8.3%	Lifetime prevalence
			3–4/10 score = problem gambling (moderate subclinical risk)	9.5%	
			** ≥ 5/10 score = pathological gambling (likely diagnosis)**	**24.6%**	
Matheson, 2021	NODS	DSM-IV pathological gambling	1–2/10 score = at-risk gambling (mild subclinical risk)	6.2%	Lifetime prevalence
			3–4/10 score = problem gambling (moderate subclinical risk)	9.3%	
			** ≥ 5/10 score = pathological gambling (likely diagnosis)**	**19.1%**	
Nower, 2015	SOGS	DSM-III/DSM-III-R pathological gambling	1–4/20 score = some problems with gambling	46.2%	Lifetime prevalence
			** ≥ 5/10 score = pathological gambling (likely diagnosis)**	**12.0%**	
Pluck, 2015	Assessment by Ricketts & Bliss	DSM-IV pathological gambling	** ≥ 5/10 score = pathological gambling (likely diagnosis)**	**31.3%**	12-months prevalence
Sharman et al., 2015	PGSI	Continuous measurement of problem gambling severity	1–4/27 score = low-risk gambling^a^	8.3%	12-months prevalence
			5–7/27 score = moderate-risk gambling^a^	3.3%	
			** ≥ 8/27 score = problem gambling**^a^	**11.6%**	
Sharman, 2016	PGSI	Continuous measurement of problem gambling severity	1–7/27 score = low-/moderate-risk gambling^a^	12.5%	12-months prevalence
			** ≥ 8/27 score = problem gambling**^a^	**23.6%**	
Wieczorek et al., 2019	PGSI	Continuous measurement of problem gambling severity	** ≥ 8/27 score = problem gambling**	**11.3%**	12-months prevalence

Bold font indicates the interpretive categories and respective prevalence estimates entered into quantitative synthesis

^a^Interpretive categories of the PGSI score according to Currie et al. 2013

C-GAM = Computerized Gambling Assessment Module; NODS = NORC Diagnostic Screen for Disorders; SOGS = South Oaks Gambling Screen; Problem Gambling Severity Index

**Fig. 2:**
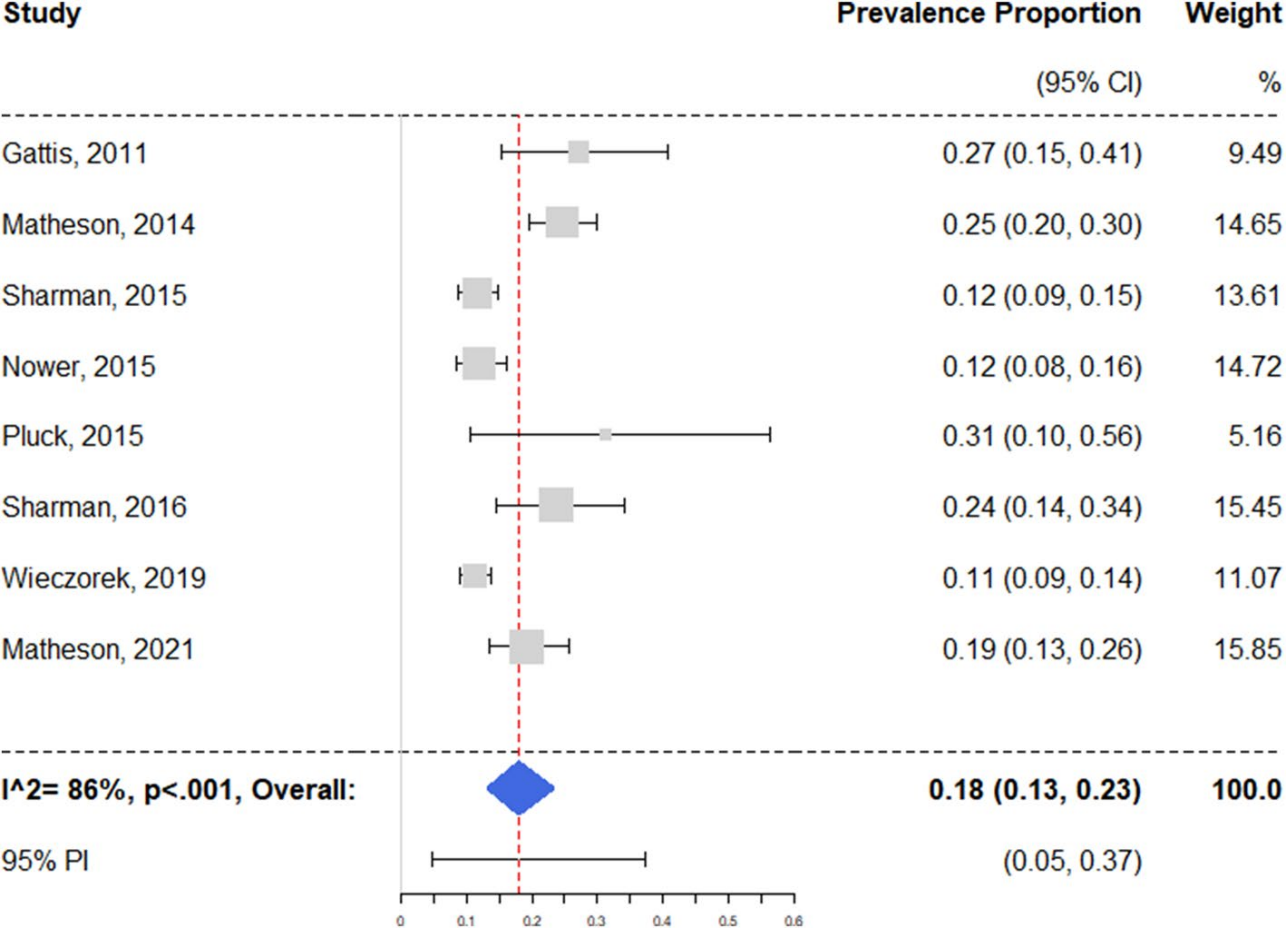
Prevalence of pathological/problem gambling. Analytical weights are from random effects meta-analysis. Legend: CI = confidence interval; PI = prediction interval

Rates of clinically relevant PG/GD were entered into a random effects meta-analysis model. The weighted mean was 18.0% (95% CI 13.2–23.3%) with a 95% PI of 4.6–37.3%. A Q-test for heterogeneity turned out significant (Q = 43.3, *p* < 0.01); the proportion of non-random variance was estimated at I^2^ = 86% (95% CI 63–97%) (see Fig. 2).

A three-level model based on the assumption that study estimates were nested within prevalence types (12-months prevalence vs. lifetime prevalence) indicated that the variance component for this additional level was at σ^2^ = 0.000. Its model fit was worse compared to the primary model (AIC_C_ 1.36 compared to -5.64).

See Table 3 for subgroup analyses. There was significant heterogeneity between subgroups when grouping by study risk of bias. The weighted mean prevalence of four studies of higher methodological quality was 13.4% (95% CI 9.0–18.5%) (see Online Resource 5).

**Table 3:** Subgroup Analyses

Grouping variable	Weighted mean (95% CI)	Q-test for Heterogeneity
***Risk of bias assessment***		**Q**_**M**_** = 9.37, *p* < 0.01**
Low Risk of Bias Studies (n = 4)	13.4% (9.0–18.5%)	
Moderate Risk of Bias Studies (n = 4)	23.3% (19.6–27.1%)	
*Prevalence Type*		Q_M_ = 0.34, *p* = 0.56
12-months Prevalence (n = 4)	16.5% (9.0–25.7%)	
Lifetime Prevalence (n = 4)	19.8% (13.4–26.9%)	
*Underlying Diagnostic Criteria*		Q_M_ = 1.64, *p* = 0.20
DSM-based Criteria (n = 5)	20.7% (14.6–27.5%)	
Other Criteria (n = 3)	14.3% (8.0–22.0%)	
*Mean Age*^†^		Q_M_ = 1.29, *p* = 0.26
Mean age > 45 years (n = 4)	21.2% (12.8–31.1%)	
Mean age < 45 years (n = 3)	14.6% (8.4%–22.1%)	
*Proportion of Female Participants*		Q_M_ = 0.00, *p* = 0.99
> 20% women (n = 3)	18.1% (10.5%–27.1%)	
< 20% women (n = 5)	18.2% (11.4%–26.0%)	

Weights are from random effects subgroup models. Bold font indicates statistically significant results

^†^One study did not report the mean age of participants

## Discussion

We conducted a systematic review and meta-analysis on the prevalence of problem gambling and gambling disorder among the homeless, including eight publications from five countries with a total of 1938 participants. Study estimates of PG/GD prevalence ranged from 11.3% to 31.3%, with a random effects weighted mean of 18.0% (95% CI 13.2–23.3%). Studies with higher methodological quality provided significantly lower prevalence estimates (13.4% (95% CI 9.0–18.5%)).

These results are in line with primary studies focussing on prevalence of PG/GD in the broader context of marginalized housing, which reported prevalence rates of 17% within users of community services in Canada (Lepage et al., 2000), 6% within clients of a Boston-based support program for homeless people with a history of substance abuse (Shaffer et al., 2002) and 12% within patients of mental health services linked to homeless hostels in Sydney (Machart et al., 2020).

The prevalence of PG/GD among people experiencing homelessness considerably exceeds rates in the general populations of the countries where the studies were conducted: 0.3% in the US (Kessler et al., 2008), similar rates in Canada and Poland (Moskalewicz et al., 2018; Rush et al., 2008), 2.6% in the UK (Gunstone et al., 2020), and 8.0% in Japan (Mori & Goto, 2020). Large population-based cross-sectional surveys identifying high rates of homelessness among patients with a PG/GD diagnosis similarly suggest an association between the two issues (Edens & Rosenheck, 2012; Moghaddam et al., 2015). There are a number of possible explanations.

First, PG/GD might negatively impact housing stability. It has been frequently reported as a key contributing factor to individuals’ pathways into homelessness (Crane et al., 2005; Laere et al., 2009; Machart et al., 2020), at least partially through financial problems and social isolation (Holdsworth & Tiyce, 2013; Sharman & D’Ardenne, 2018). Second, homelessness might reversely be a factor contributing to or at least maintaining PG/GD. Gambling behaviour might function as a coping mechanism in housing exclusion, providing distraction, a sense of meaning or even just a warm place to stay, or be motivated by hopes of drastically altering ones living situation through a “big win” (Holdsworth & Tiyce, 2013; Sharman & D’Ardenne, 2018). Third, the relationship between homelessness and PG/GD might be to a certain degree confounded by shared risk factors, such as childhood abuse, relationship breakdown, violent victimization or criminal conviction (Nilsson et al., 2019; Roberts et al., 2017).

Special attention should be paid to the complex interconnection of both homelessness and gambling problems with substance use disorders (Fazel et al., 2008; Landon et al., 2021; Lorains et al., 2011). GD and substance use disorders are characterized by common underlying neurobiological and genetic factors, pointing toward a shared vulnerability (Wareham & Potenza, 2010). The relationship of homelessness and substance use disorders has been theorized to be bidirectional (Schreiter et al., 2020), but might also to a high degree be mediated by common individual risk factors (McVicar et al., 2015).

However, social and clinical support services addressing people experiencing homelessness should be developed to manage high rates of PG/GD. Only a small share of people with PG actively seek treatment (Slutske, 2006), which might be particularly the case for people in homelessness (e.g. competing priorities) (Holdsworth & Tiyce, 2013; Sharman & D’Ardenne, 2018). This highlights the importance of practitioners being aware of the importance of PG/GD and the use of effective diagnostic tools for early detection, which, as limited qualitative data suggests, is currently often not the case (Landon et al., 2021). Useful materials that may assist service providers have been developed in a UK-based pilot study, including an information sheet, a screening tool tailored to people in homelessness and a resource sheet providing immediate advice and contact information of available support services, but require validation in larger samples and other languages (Sharman & D’Ardenne, 2018). With its advantageous psychometric properties, the PGSI, defining caseness at a score of 8 or above, might also be a useful screening instrument (Orford et al., 2010).

The social and health needs of people experiencing homelessness and PG/GD need to be addressed with integrated approaches, accounting for their multidimensional needs (Landon et al., 2021). In settings where more long-term treatments are not feasible, brief motivational interventions can already have lasting positive effects (Petry et al., 2008).

Further investigations into the prevalence of PG/GD in homeless populations are indicated. Prevalence rates among the homeless might strongly depend on localized factors like the social support system in cases of homelessness and mental health care services for PG/GD as well as gambling legislations. Therefore, researchers and practitioners would benefit from data as specific to their respective settings as possible. Future researchers should take care to recruit large enough samples and optimize their methodology with representative sampling methods and transparent participation rates to avoid risk of bias. So far, most of the utilized screening instruments relied on dated editions of DSM and it remains to be seen how the criteria of DSM-5 impact prevalence estimates. It has been argued that increased rates are to be expected particularly in high-risk populations like the homeless (Rash & Petry, 2016). Future researchers should focus on GD as a preferred outcome. Furthermore, at this point, research into specific interventions for PG/GD for homeless individuals is still lacking.

Notable limitations include differences of utilized screening instruments and prevalence types (past year vs. lifetime assessment) between studies, restricting comparability. Both factors have been described as some of the most important methodological characteristics to influence PG prevalence estimates (Williams et al., 2012). Subgroup analyses based on these characteristics did not suggest significant differences, but this might be due to the small sample size. With eight publications from five countries being eligible to this review, generalisability of the results is limited. As the wide prediction interval (4.6–37.3%) indicates, results of possible additional study samples could be considerably dispersed. Investigating more population level predictors for PG/GD prevalence rates, possibly by meta-regression models, was not performed due to sparse reports on sample characteristics in primary studies and the overall small sample size. We addressed the substantial amount between-study heterogeneity (I^2^ = 86%) with subgroup-analysis on low risk of bias studies reporting significantly lower prevalence rates.

In conclusion, we found that at least one in ten people in homelessness are affected by PG/GD. Our data on the one hand elucidates questions of methodology in future research in this field like sampling procedures, the need for standardized instruments and sample size. On the other hand, our results identify future fields of interest, especially individual predictors of PG/GD in the homeless and prevalence in different regions as well as affecting factors like gambling legislature.

## Supplementary Information

Below is the link to the electronic supplementary material.

Online Resource 1 PRISMA Checklist (DOCX 27 kb)

Online Resource 2 Search Strings (DOCX 13 kb)

Online Resource 3 Excluded Studies (DOCX 15 kb)

Online Resource 4 Risk of Bias Assessment (DOCX 15 kb)

Online Resource 5 Subgroup Analysis by Risk of Bias. Analytical weights are from random effectsmeta-analyses for each subgroup separately. Legend: CI = confidence interval; PI = predictioninterval. (TIFF 2257 kb)

## Data Availability

Data sharing is not applicable to this article as no new data were created or analysed in this study.
