# Supplementary material for: The Prevalence of Problem Gambling and Gambling Disorder Among Homeless People: A Systematic Review And Meta-Analysis
Source: J Gambl Stud. 2022 Jul 19;39(2):467–82. doi: 10.1007/s10899-022-10140-8 (PMC10175321; doi:10.1007/s10899-022-10140-8)
Supplement: Supplementary file 1 — PRISMA Checklist (DOCX 27 kb) [file 10899_2022_10140_MOESM1_ESM.docx]

| **Section/topic** | **#** | **Checklist item** | **Reported on page #** |
| --- | --- | --- | --- |
| **TITLE** | | |  |
| Title | 1 | Identify the report as a systematic review, meta-analysis, or both. | Title:  “THE PREVALENCE OF PROBLEM GAMBLING AND GAMBLING DISORDER AMONG HOMELESS PEOPLE: A SYSTEMATIC REVIEW AND META-ANALYSIS” |
| **ABSTRACT** | | |  |
| Structured summary | 2 | Provide a structured summary including, as applicable: background; objectives; data sources; study eligibility criteria, participants, and interventions; study appraisal and synthesis methods; results; limitations; conclusions and implications of key findings; systematic review registration number. | Abstract:  “Background and Aims  Gambling problems are often associated with homelessness and linked to elevated psychiatric morbidity and homelessness chronicity. We performed a systematic review and meta-analysis on prevalence rates of problem gambling and gambling disorder in homeless people.  Methods (…)” |
| **INTRODUCTION** | | |  |
| Rationale | 3 | Describe the rationale for the review in the context of what is already known. | Introduction, paragraph 6:  “Precise estimates on the prevalence of PG/GD among the homeless are important to inform service development and evidence-based policy. Several publications have narratively reviewed literature on the prevalence of PG/GD in people experiencing homelessness (20,23,24), but there are no systematic reviews to our knowledge.” |
| Objectives | 4 | Provide an explicit statement of questions being addressed with reference to participants, interventions, comparisons, outcomes, and study design (PICOS). | Introduction, paragraph 6:  “We therefore aim to provide a systematic review and meta-analysis on the prevalence of PG and GD in homeless populations.” |
| **METHODS** | | |  |
| Protocol and registration | 5 | Indicate if a review protocol exists, if and where it can be accessed (e.g., Web address), and, if available, provide registration information including registration number. | Methods, paragraph 1:  “The protocol for this review was registered at PROSPERO (registration ID CRD42021233670). The authors followed the PRISMA statement (Preferred Reporting Items for Systematic Reviews and Meta-Analyses (25), see Table S1).” |
| Eligibility criteria | 6 | Specify study characteristics (e.g., PICOS, length of follow-up) and report characteristics (e.g., years considered, language, publication status) used as criteria for eligibility, giving rationale. | Methods, paragraphs 2-4:  “Eligibility Criteria  We sought to identify primary studies that could provide prevalence estimates of PG/GD in homeless samples in online scientific data bases. Studies had to meet the following inclusion criteria to be included in the review:  A) A prevalence estimate (12-months prevalence or lifetime prevalence) of PG/GD was determined.  B) A separate sample of exclusively and reliably homeless individuals was included.  C) Participants were individually examined for PG/GD using a standardized diagnostic instrument.  Studies which sampled specific subpopulations not representative for the homeless population as a whole (i.e., exclusively homeless persons with mental disorders, selected age bands etc.) were to be excluded.” |
| Information sources | 7 | Describe all information sources (e.g., databases with dates of coverage, contact with study authors to identify additional studies) in the search and date last searched. | Methods, paragraph 5:  “In order to identify eligible records, Medline via PubMed, Embase via OvidSP and PsycInfo via EBSCOhost were searched by specifically formulated entries containing key words associated with homelessness and gambling (see Table S2). Additionally, we screened the reference lists of included and other major publications for relevant studies.” |
| Search | 8 | Present full electronic search strategy for at least one database, including any limits used, such that it could be repeated. | Methods, paragraph 5:  “In order to identify eligible records, Medline via PubMed, Embase via OvidSP and PsycInfo via EBSCOhost were searched by specifically formulated entries containing key words associated with homelessness and gambling (see Table S2). Additionally, we screened the reference lists of included and other major publications for relevant studies. No restrictions on publication language were applied. Records published between the inception of data bases and 4th of May 2021 were included.” |
| Study selection | 9 | State the process for selecting studies (i.e., screening, eligibility, included in systematic review, and, if applicable, included in the meta-analysis). | Methods, paragraph 6:  “Search results were independently scanned for eligible articles by two researchers. Differences in screening results were resolved in discussion.“ |
| Data collection process | 10 | Describe method of data extraction from reports (e.g., piloted forms, independently, in duplicate) and any processes for obtaining and confirming data from investigators. | Methods, paragraph 7:  “Data from included studies for study location, years of study conduct, assessment used in diagnosing PG/GD, recruitment strategy, sampling method, information regarding psychiatric morbidity, mean age, gender distribution, sample size and number of detected cases of PG/GD was extracted. In cases of missing information, authors of primary studies were contacted to provide additional data.”  Methods, paragraph 10:  “Both data extraction and quality evaluation were carried out by two researchers independently from one another, discussing diverging results afterwards.” |
| Data items | 11 | List and define all variables for which data were sought (e.g., PICOS, funding sources) and any assumptions and simplifications made. | Methods, paragraph 7:  ““Data from included studies for study location, years of study conduct, assessment used in diagnosing PG/GD, recruitment strategy, sampling method, information regarding psychiatric morbidity, mean age, gender distribution, sample size and number of detected cases of PG/GD was extracted.” |
| Risk of bias in individual studies | 12 | Describe methods used for assessing risk of bias of individual studies (including specification of whether this was done at the study or outcome level), and how this information is to be used in any data synthesis. | Methods, paragraph 9:  “Included studies were evaluated regarding risk of bias by a standardized assessment tool (26). Each item was individually evaluated. For the summary item, we rated studies as low risk of bias when eight or more items out of 10 items indicated “low risk”, any others as moderate risk of bias.” |
| Summary measures | 13 | State the principal summary measures (e.g., risk ratio, difference in means). | Methods, paragraph 11:  “Prevalence estimates corresponding to clinically relevant PG/GD were entered into a meta-analytical model.” |
| Synthesis of results | 14 | Describe the methods of handling data and combining results of studies, if done, including measures of consistency (e.g., I^2^) for each meta-analysis. | Methods, paragraph 12:  “All statistical analyses were carried out in in R, version 4.0.4 (27), using the package ‘metafor’, version 2.4-0 (28). A Freeman Turkey double arcsine transformation was applied to the prevalence estimates (29), so variance instability could be avoided (30). We calculated random effects models, estimating the variance by the Paule-Mandel method (31). A 95% Wald-type confidence interval (CI) was computed around the random effects weighted mean, as well as a 95% prediction interval (PI), the latter by a method which accounts for the model variance to be an estimated value ((32), expression 12). A Q-test for heterogeneity was conducted and the I2 statistic was computed (33).” |

| **Section/topic** | **#** | **Checklist item** | **Reported on page #** |
| --- | --- | --- | --- |
| Risk of bias across studies | 15 | Specify any assessment of risk of bias that may affect the cumulative evidence (e.g., publication bias, selective reporting within studies). | Methods, paragraph 14:  “To examine the impact of methodological characteristics, we conducted subgroup analyses, grouping studies by prevalence type (lifetime vs. past-year prevalence), PG/GD criteria (DSM-based vs. not DSM-based), overall risk of bias (low risk of bias vs. moderate risk of bias), sample mean age (>45years vs. <45years) and proportion of female participants (>20% vs. <20%). Random effects weighted means and 95% CIs were calculated for each group separately and the between-groups heterogeneity was assessed by a Q-test. “ |
| Additional analyses | 16 | Describe methods of additional analyses (e.g., sensitivity or subgroup analyses, meta-regression), if done, indicating which were pre-specified. | Methods, paragraph 13-14:  For a secondary analysis, we constructed a three-level meta-analytic model for the same data, using the ‘metafor::rma.mv’ function. The underlying assumption was that the 12-months prevalence rates and lifetime prevalence rates included in the analysis might constitute slightly different effect sizes, introducing a dependency (study estimates being “nested” within the prevalence types) which might lead to an underestimation of the model heterogeneity. A three-level model has an additional layer integrated into its structure to account for clustered data like this (34). The fit of this secondary model was compared to the primary one with the ‘metafor::anova’ function by the Akaike criterion corrected for small samples (AICC).  To examine the impact of methodological characteristics, we conducted subgroup analyses, grouping studies by prevalence type (lifetime vs. past-year prevalence), PG/GD criteria (DSM-based vs. not DSM-based), overall risk of bias (low risk of bias vs. moderate risk of bias), sample mean age (>45years vs. <45years) and proportion of female participants (>20% vs. <20%). Random effects weighted means and 95% CIs were calculated for each group separately and the between-groups heterogeneity was assessed by a Q-test.” |
| **RESULTS** | | |  |
| Study selection | 17 | Give numbers of studies screened, assessed for eligibility, and included in the review, with reasons for exclusions at each stage, ideally with a flow diagram. | Results, paragraph 1:  “The database search entries returned 310 distinct records after duplicates were removed (see Fig. 1). Eight publications were found to be eligible (35–42) (for information on articles rejected in full-text screening see Table S3). They were published between 2011 and 2021 and conducted in five different countries: Japan (39), Poland (42) and two each in Canada (36,37), the US (35,38) and the UK (40,41).” |
| Study characteristics | 18 | For each study, present characteristics for which data were extracted (e.g., study size, PICOS, follow-up period) and provide the citations. | Results, paragraph 2:  “Data on a total of 1938 homeless individuals was included by these surveys. For 1527 (77.0%) participants, information on gender was provided, identifying 1179 (77.2%) as male and 348 (22.8%) as female (35,36,38–42). A mean age of 46 years (38–42) was reported in 1213 (61.1%) participants . See Table 1 for additional study characteristics.” |
| Risk of bias within studies | 19 | Present data on risk of bias of each study and, if available, any outcome level assessment (see item 12). | Results, paragraph 2:  “In quality assessment, four studies were rated as low risk of bias and four as moderate risk of bias (see Table 2).” |
| Results of individual studies | 20 | For all outcomes considered (benefits or harms), present, for each study: (a) simple summary data for each intervention group (b) effect estimates and confidence intervals, ideally with a forest plot. | Results, paragraphs 3-8 |
| Synthesis of results | 21 | Present results of each meta-analysis done, including confidence intervals and measures of consistency. | Results, paragraph 6:  “Rates of clinically relevant PG/GD were entered into a random effects meta-analysis model. The weighted mean was 18.0% (95% CI 13.2% - 23.3%) with a 95% PI of 4.6% - 37.3%. A Q-test for heterogeneity turned out significant (Q=43.3, p<0.01); the proportion of non-random variance was estimated at I2=86% (95% CI 63% - 97%) (see Fig. 2).” |
| Risk of bias across studies | 22 | Present results of any assessment of risk of bias across studies (see Item 15). | Results, paragraph 8:  “See Table 4 for subgroup analyses. There was significant heterogeneity between subgroups when grouping by study risk of bias. The weighted mean prevalence of four studies of higher methodological quality was 13.4% (95% CI 9.0% - 18.5%) (see Fig. 3).” |
| Additional analysis | 23 | Give results of additional analyses, if done (e.g., sensitivity or subgroup analyses, meta-regression [see Item 16]). | Results, paragraph 7:  “A three-level model based on the assumption that study estimates were nested within prevalence types (12-months prevalence vs. lifetime prevalence) indicated that the variance component for this additional level was at σ2=0.000. Its model fit was worse compared to the primary model (AICC 1.36 compared to -5.64).”  Results, paragraph 8:  “See Table 4 for subgroup analyses. There was significant heterogeneity between subgroups when grouping by study risk of bias. The weighted mean prevalence of four studies of higher methodological quality was 13.4% (95% CI 9.0% - 18.5%) (see Fig. 3).” |
| **DISCUSSION** | | |  |
| Summary of evidence | 24 | Summarize the main findings including the strength of evidence for each main outcome; consider their relevance to key groups (e.g., healthcare providers, users, and policy makers). | Discussion, paragraphs 1-10:  “We conducted a systematic review and meta-analysis on the prevalence of problem gambling and gambling disorder among the homeless, including eight publications from five countries with a total of 1938 participants. Study estimates ranged from 11.3% to 31.3%, with a random effects weighted mean of 18.0% (95% CI 13.2% - 23.3%). Studies with higher methodological quality provided significantly lower prevalence estimates (13.4% (95% CI 9.0% - 18.5%)).  (…)” |
| Limitations | 25 | Discuss limitations at study and outcome level (e.g., risk of bias), and at review-level (e.g., incomplete retrieval of identified research, reporting bias). | Discussion, paragraph 11:  “Limitations  Notable limitations include differences of utilized screening instruments and prevalence types (past year vs. lifetime assessment) between studies, restricting comparability. Both factors have been described as some of the most important methodological characteristics to influence PG prevalence estimates (71). Subgroup analyses based on these characteristics did not suggest significant differences, but this might be due to the small sample size. Only eight publications from five countries being eligible to this review limits the generalisability of the results considerably. As the wide prediction interval (4.6% - 37.3%) indicates, results of possible additional study samples could be heavily dispersed. Investigating more population level predictors for PG/GD prevalence rates, possibly by meta-regression models, was not feasible due to sparse reports on sample characteristics in primary studies and the overall small sample size. Therefore, aside from low risk of bias studies reporting significantly lower prevalence rates, the substantial amount between-study heterogeneity (I2=86%) remains mostly unexplained.” |
| Conclusions | 26 | Provide a general interpretation of the results in the context of other evidence, and implications for future research. | Discussion, paragraph 12:  “Despite the limitations of the available research, we found that at least one in ten people in homelessness have PG/GD. Because of the small number of studies from few countries, further research is required to better understand individual predictors of PG/GD in the homeless and prevalence in different regions of the world. Additionally, future research should focus on how care providers might be able to effectively detect PG/GD among their homeless clients and offer treatment.” |
| **FUNDING** | | |  |
| Funding | 27 | Describe sources of funding for the systematic review and other support (e.g., supply of data); role of funders for the systematic review. | Funding:  “The authors of this systematic review and meta-analysis did not receive any funds for this research.” |

*From:*  Moher D, Liberati A, Tetzlaff J, Altman DG, The PRISMA Group (2009). Preferred Reporting Items for Systematic Reviews and Meta-Analyses: The PRISMA Statement. PLoS Med 6(6): e1000097. doi:10.1371/journal.pmed1000097
