# Supplementary material for: The Prevalence of Problem Gambling and Gambling Disorder Among Homeless People: A Systematic Review And Meta-Analysis
Source: J Gambl Stud. 2022 Jul 19;39(2):467–82. doi: 10.1007/s10899-022-10140-8 (PMC10175321; doi:10.1007/s10899-022-10140-8)
Supplement: Supplementary file 2 — Search Strings (DOCX 13 kb) [file 10899_2022_10140_MOESM2_ESM.docx]

| **Table S2 – Search Strings** | | |
| --- | --- | --- |
| **Data Base** | **Search Query** | **Results (n)** |
| Medline via PubMed | *(gambling OR betting OR "Gambling"[Mesh]) AND (homeless* OR „Homeless Persons"[MeSH] OR roofless* OR shelter* OR "Housing"[Mesh] OR housing)*  Assessed on 04/05/2021, 11:00 | 240 |
| Embase via OvidSP | *1. exp pathological gambling/ or exp gambling/ or gambling.mp.*  *2. betting.mp.*  *3. 1 or 2*  *4. homeless.mp. or exp homeless person/*  *5. homelessness.mp. or exp homelessness/*  *6. roofless.mp.*  *7. shelter.mp.*  *8. exp housing/ or housing.mp.*  *9. 4 or 5 or 6 or 7 or 8*  *10. 3 and 9*  Assessed on 04/05/2021, 11:00 | 72 |
| PsycINFO via EBSCOhost | *(gambling OR betting OR DE "Gambling" OR DE "Gambling Disorder") AND (homeless* OR (DE “Homeless”) OR roofless* OR shelter* OR (DE “Shelters”) OR housing OR (DE”housing”))*  Assessed on 04/05/2021, 11:00 | 90 |
