# Supplementary material for: The Prevalence of Problem Gambling and Gambling Disorder Among Homeless People: A Systematic Review And Meta-Analysis
Source: J Gambl Stud. 2022 Jul 19;39(2):467–82. doi: 10.1007/s10899-022-10140-8 (PMC10175321; doi:10.1007/s10899-022-10140-8)
Supplement: Supplementary file 3 — Excluded Studies (DOCX 15 kb) [file 10899_2022_10140_MOESM3_ESM.docx]

| Table S3. Records Excluded in Full-Text Screening | | |
| --- | --- | --- |
| # | Study | Reasons for Exclusion |
| 1. | Heffron WA, Skipper BJ, Lambert L. Health and lifestyle issues as risk factors for homelessness. J Am Board Fam Pract. 1997;10(1):6–12. | Incl. C: Only self-reported history of gambling problems was assessed.  Excl. A: Only patients of a health care facility were recruited. |
| 2. | Lepage C, Ladouceur R, Jacques C. Prevalence of Problem Gambling Among Community Service Users. Community Ment Health J. 2000;36(6):597–601. | Incl. B: Most participants were not homeless |
| 3. | Machart T, Cooper L, Jones N, Nielssen A, Doughty E, Staples L, et al. Problem gambling among homeless clinic attenders. Australas Psychiatry. 2020 Feb;28(1):91–4. | Incl. C: Only self-report of gambling as a reason for homelessness was assessed.  Excl. A: Only persons who presented to a mental health clinic were included. |
| 4. | Nielssen OB, Stone W, Jones NM, Challis S, Nielssen A, Elliott G, et al. Characteristics of people attending psychiatric clinics in inner sydney homeless hostels. Med J Aust. 2018;208(4):169–73. | Incl. C: Type of assessment for gambling problems not reported.  Excl. A: Only persons who presented to a mental health clinic were included. |
| 5. | Shaffer HJ, Freed CR, Healea D. Gambling disorders among homeless persons with substance use disorders seeking treatment at a community center. Psychiatr Serv. 2002 Sep;53(9):1112–7. | Excl. A: Only homeless individuals with current or past substance use disorder |
| 6. | Yamamoto M, Horita R, Sado T, Nishio A. Non-communicable Disease among Homeless Men in Nagoya, Japan: Relationship between Metabolic Abnormalities and Sociodemographic Backgrounds. Intern Med. 2020;59(9):1155–62. | Incl. A: Only self-report of gambling habit, no assessment of problem gambling/gambling disorder |
| Incl. A = A prevalence estimate of problem gambling or gambling disorder was determined.  Incl. B = A separate sample of exclusively and reliably homeless individuals was included.  Incl. C = Participants were individually examined for problem gambling or gambling disorder using a standardized screening instrument.  Excl. A = Studies which sampled specific sub-populations were excluded. | | |
