# Supplementary material for: The Prevalence of Problem Gambling and Gambling Disorder Among Homeless People: A Systematic Review And Meta-Analysis
Source: J Gambl Stud. 2022 Jul 19;39(2):467–82. doi: 10.1007/s10899-022-10140-8 (PMC10175321; doi:10.1007/s10899-022-10140-8)
Supplement: Supplementary file 4 — Risk of Bias Assessment (DOCX 15 kb) [file 10899_2022_10140_MOESM4_ESM.docx]

| **Online Resource 4. Risk of Bias Assessment** | | | | | | | | | | |
| --- | --- | --- | --- | --- | --- | --- | --- | --- | --- | --- |
| **Study** | **Q2** | **Q3** | **Q4** | **Q5** | **Q6** | **Q7** | **Q8** | **Q9** | **Q10** | **Summary Item** |
| Gattis, 2011 | No | No | No | Yes | Yes | Yes | Yes | No | Yes | Moderate Risk |
| Matheson, 2014 | Yes | No | No | Yes | Yes | Yes | Yes | No | Yes | Moderate Risk |
| Matheson, 2021 | Yes | No | No | Yes | Yes | Yes | Yes | No | Yes | Moderate Risk |
| Nower, 2015 | Yes | Yes | Yes | Yes | Yes | Yes | Yes | No | Yes | Low Risk |
| Pluck, 2015 | No | No | Yes | Yes | Yes | No | Yes | Yes | Yes | Moderate Risk |
| Sharman, 2014 | Yes | No | No | Yes | Yes | Yes | Yes | Yes | Yes | Low Risk |
| Sharman, 2016 | Yes | No | No | Yes | Yes | Yes | Yes | Yes | Yes | Low Risk |
| Wiezcorek, 2020 | Yes | Yes | No | Yes | Yes | Yes | Yes | Yes | Yes | Low Risk |
| Q1: (*Disregarded, not relevant for the aim of this review*)  Q2: Was the sampling frame a true or close representation of the target population?  Q3: Was some form of random selection used to select the sample, OR, was a census undertaken?  Q4: Was the likelihood of non-response bias minimal?  Q5: Were data collected directly from the subjects (as opposed to a proxy)?  Q6: Was an acceptable case definition used in the study?  Q7: Was the study instrument that measured the parameter of interest (e.g. prevalence of low back pain) shown to have reliability and validity (if necessary)? Q8: Was the same mode of data collection used for all subjects? Q9: Was the length of the shortest prevalence period for the parameter of interest appropriate?  Q10: Were the numerator(s) and denominator(s) for the parameter of interest appropriate? | | | | | | | | | | |
