# Supplementary figures and images for: The Prevalence of Problem Gambling and Gambling Disorder Among Homeless People: A Systematic Review And Meta-Analysis
Source: J Gambl Stud. 2022 Jul 19;39(2):467–82. doi: 10.1007/s10899-022-10140-8 (PMC10175321; doi:10.1007/s10899-022-10140-8)

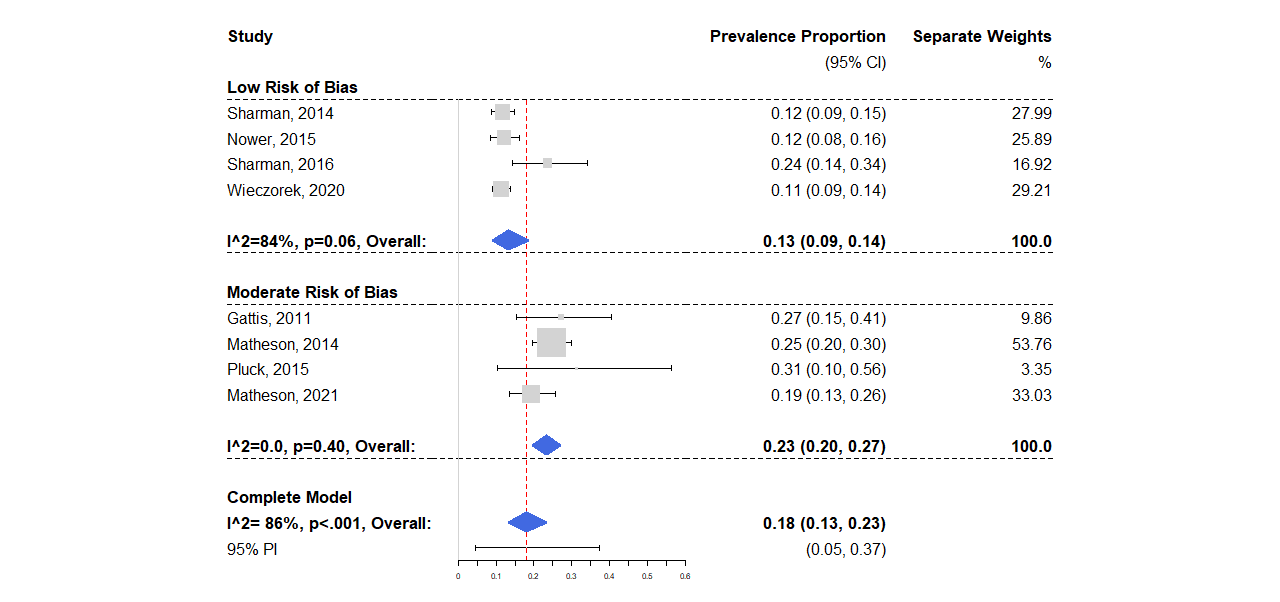

Supplement: Supplementary file 5 — Subgroup Analysis by Risk of Bias. Analytical weights are from random effectsmeta-analyses for each subgroup separately. Legend: CI = confidence interval; PI = predictioninterval. (TIFF 2257 kb) [file 10899_2022_10140_MOESM5_ESM.tiff]
